# Thyroid Disease–Induced Hepatic Dysfunction: A Clinical Puzzle

**DOI:** 10.14309/crj.0000000000000555

**Published:** 2021-04-14

**Authors:** Chimaobi M. Anugwom, Thomas M. Leventhal

**Affiliations:** 1 Division of Gastroenterology, Hepatology, and Nutrition, University of Minnesota, Minneapolis, MN

## Abstract

The diagnostic evaluation of an individual with clinical and laboratory evidence of thyroid dysfunction in the setting of acute liver injury is crucial. There is a complex relationship between the thyroid and the liver, and so, it requires a careful elucidation of the inciting disease process before instituting a treatment plan. We discuss a patient who had presented with coagulopathy, encephalopathy, and laboratory evidence of acute liver injury, hence adjudged to have developed drug-induced acute liver failure and transferred for liver transplant evaluation. She was found to have liver dysfunction from uncontrolled thyroid disease, with immediate and rapid improvement after controlling severe hyperthyroidism.

## INTRODUCTION

There is a well-known physiologic and pathologic relationship that exists between the liver and the thyroid gland. For this reason, the diagnostic evaluation of an individual with clinical and laboratory evidence of thyroid dysfunction in the setting of acute liver injury is crucial. It requires a careful elucidation of the inciting disease process before instituting a treatment plan. Here, we present a patient, adjudged to have developed drug-induced acute liver failure, who was found to have liver dysfunction from uncontrolled thyroid disease.

## CASE REPORT

**Figure 1.**
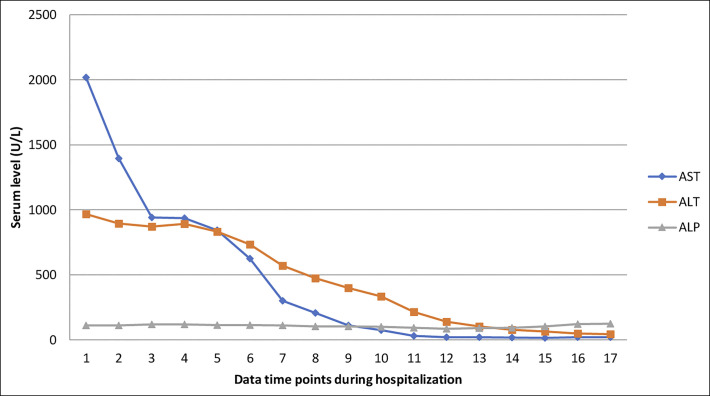
Trend of liver enzymes during hospitalization.

**Figure 2.**
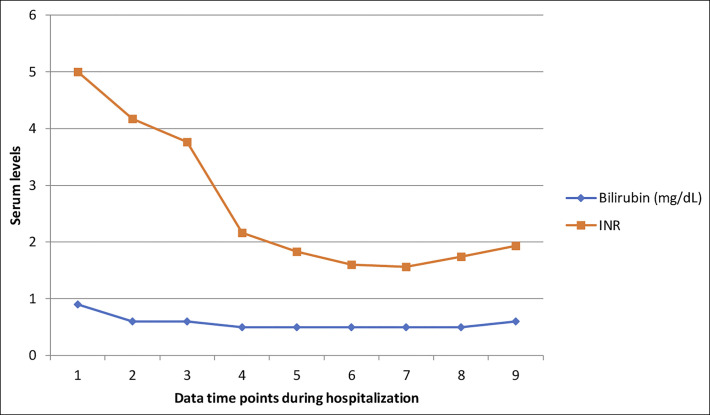
Trend of total bilirubin and international normalized ratio during hospitalization. INR, international normalized ratio.

A 66-year-old woman with type 2 diabetes mellitus, atrial fibrillation on warfarin, as well as a recent diagnosis of Graves disease (positive thyroid-stimulating immunoglobulins and radioactive iodine uptake of 99%) was transferred to our facility with a diagnosis of drug-induced liver injury with resultant acute liver failure. She had initially presented with several weeks of malaise after being started on methimazole for her Graves disease, and she had taken this medication for only 4 days. Initial laboratory findings included markedly elevated liver transaminases, and her international normalized ratio (INR) was also high (Figures 1 and 2). Although she was being assessed and stabilized in the emergency department, she developed rapid deterioration of her mental status with lethargy, weakness, and progressed to frank confusion and somnolence. This led to urgent endotracheal intubation to ensure airway protection. To evaluate her confusion, an magnetic resonance imaging of the brain was obtained which did not show any acute intracranial pathology but noted findings suggesting chronic small vessel ischemic disease and mild diffuse cerebral atrophy. Based on the findings of coagulopathy and progressive encephalopathy in the setting of acute liver injury, she was diagnosed as having acute liver failure, N-acetylcysteine was started, and she was transferred to our transplant center for evaluation for liver transplantation.

**Figure 3.**
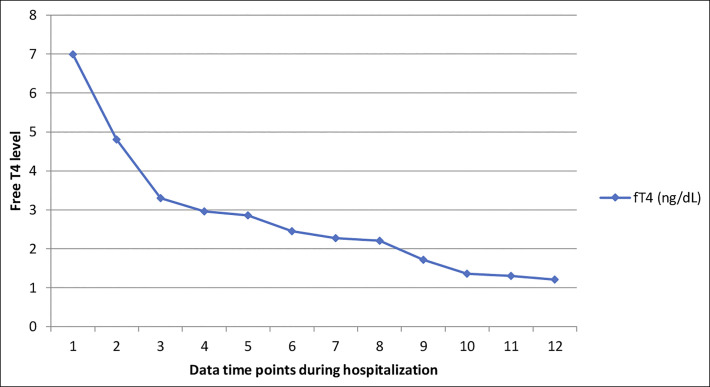
Trend of serum-free thyroxine during hospitalization.

Her medications were reviewed thoroughly, and the possibility of liver injury secondary to methimazole was entertained, as this was a new medication, so it was initially discontinued. On evaluation, she was afebrile but in atrial fibrillation with a heart rate of 118. She was initially hypotensive at 86/49 mm Hg. She was believed to have a Burch-Wartofsky Point Scale of 55, thus highly suggestive of severe thyrotoxicosis/thyroid storm. A comprehensive etiological workup was undertaken which yielded a negative antinuclear antibody and anti-smooth muscle antibody; negative viral serology for hepatitis A, B, and C, human immunodeficiency virus, and herpes simplex virus. She had a normal creatine kinase, and her serum acetaminophen and salicylate levels were below detectable range. She underwent a urine toxicology screen that was negative for alcohol and other drugs. A complete abdominal ultrasound with Dopplers was notable for a grossly normal liver with mild steatosis but no structural or vascular abnormalities. A portable chest radiograph noted mild pulmonary edema and cardiomegaly. A transthoracic echocardiogram was obtained as well, and this was significant for a left ventricular ejection fraction of 36%, with an enlarged right ventricle, moderately reduced right ventricular function, and a dilated inferior vena cava. Her thyroid function tests revealed undetectable thyroid-stimulating hormone and elevated free thyroxine hormones (free T4 and T3) (Figure 3). A diagnosis of thyrotoxicosis was made with resultant congestive cardiac failure and subsequent congestive hepatopathy. She was started on high-dose intravenous methylprednisolone and restarted on her methimazole. She improved clinically and had laboratory normalization of her INR and liver tests. She was ultimately discharged, clinically stable, to follow-up in the outpatient endocrinology clinic.

## DISCUSSION

Hepatocyte activity is dependent on the regulatory function of thyroid hormones, and the liver plays a major role in the metabolism of these hormones, consequently affecting the systemic endocrine effects of the thyroid gland.^1,2^ The pathologic relationship between the liver and the thyroid gland has many facets because a myriad of liver diseases have been linked with thyroid disease and vice versa. Autoimmune hepatitis and primary biliary cholangitis have both been associated with autoimmune thyroiditis and Graves disease.^3,4^ Furthermore, hepatitis C virus (HCV) infection has also been linked with thyroid dysfunction as well—the presence of HCV seropositivity has been documented to coexist with development of thyroid autoimmunity and hypothyroidism.^5^ In addition, before the era of direct-acting antivirals in the treatment of HCV, interferon has been postulated to induce a direct toxic effect on thyroid follicular cells as well as cause autoimmune dysfunction.^6,7^ Our patient was therefore evaluated for primary liver diseases that share known associations with Graves disease and was negative for HCV infection, primary biliary cholangitis, and autoimmune hepatitis.

Thyroid dysfunction is treated with medications that may also cause hepatocellular or cholestatic liver injury. Propylthiouracil and methimazole have been known to cause transient, asymptomatic elevations in serum aminotransferase levels in the first 3 months of therapy, and the pattern of injury is typically hepatocellular. Cases of moderately severe cholestatic or mixed hepatitis have also been described and sometimes can progress to acute liver failure, which has necessitated liver transplantation or lead to death.^8^ The putative mechanism of this type of liver injury is an immunological reaction to a metabolic by-product of the medication.^8–10^ The possibility of liver injury secondary to methimazole was entertained, leading to stoppage of the medication on initial presentation. Our patient had been on this medication for less than 5 days, and it was hypothesized that the likelihood of drug-induced liver injury was low in this case.

Atrial fibrillation with a rapid ventricular rate on admission, a transthoracic echocardiogram was notable for significant right and left heart failure, with ventricular enlargement and decreased ejection fraction. The cardiovascular effect of thyroid dysfunction, as was apparent in our patient, has been shown to occur and may lead to liver dysfunction in multiple ways. Increased myocellular contraction, increased oxygen demand, increased preload, and decreased afterload may lead to profound tachyarrhythmia and, at times, atrial fibrillation, cardiomyopathy, and in severe cases, valvulopathy.^11–13^ This may result in poor forward flow and cardiogenic shock presenting as ischemic hepatitis. Severe congestive heart failure with impaired venous return, if long-standing, may result in passive hepatic congestion and subsequent centrilobular necrosis with marked abnormalities in serum transaminases, bilirubin, as well as the development of hepatomegaly and coagulopathy. Histological evidence of hepatic fibrosis may become apparent as congestion becomes more marked and prolonged.^14,15^ Thyrotoxicosis is treated with antithyroid medications such as methimazole, as well as beta-blockade. Patients are also commenced on intravenous corticosteroids. Corticosteroids inhibit peripheral conversion of T4 into T3 and have been shown to improve outcomes in patients with thyroid storm. Varying degrees of adrenal axis dysfunction may coexist in these patients, and so, steroids are used until resolution of the toxic state.^16^ Thyroidectomy is occasionally used in the cases that are refractory to medication but is associated with a risk of storm exacerbation if preoperative thyroid hormone levels are high.^17^

In our patient, methimazole was restarted given its low likelihood of being the cause of her liver injury. The patient clinically improved with correction of the severe thyroid abnormalities, with rapid normalization in coagulopathy, liver enzymes, and cardiac function (on follow-up transthoracic echocardiogram). In conclusion, our patient developed severe acute liver injury from uncontrolled Graves disease. The combination of centrilobular necrosis as shown by the elevated INR, aspartate aminotransferase, and alanine aminotransferase with evidence of biventricular heart failure in a patient with seemingly uncontrolled hyperthyroidism was suggestive of the diagnosis. Complete improvement with reduction of fT4 using methimazole was also supportive of this diagnosis. Liver injury is a disease process that requires careful investigation because management options vary depending on the underlying etiology. Determining the inciting process is crucial for guiding not only treatment, but for assessing need of resource utilization, including potential for liver transplantation.

## DISCLOSURES

Author contributions: Both authors contributed equally to this manuscript. TM Leventhal is the article guarantor.

Financial disclosure: None to report.

Informed consent could not be obtained from the patient despite several attempts. All identifying information has been removed from this case report to protect patient privacy.
